# Expression of Concern: HiSV: A control-free method for structural variation detection from Hi-C data

**DOI:** 10.1371/journal.pcbi.1013016

**Published:** 2025-04-17

**Authors:** The PLOS Computational Biology Editors

Concerns were raised about this article [1] after the uncorrected proof version was published. PLOS held the article at the uncorrected proof stage while following up on the concerns.

The issues raised included concerns regarding unsupported claims, errors in the code on GitHub [2], and unavailability of the underlying data. In response, the corresponding author revised the code and documentation on GitHub [2], and updated the article to address the following:

- The datasets used for analysis and information about the datasets were added as Supporting Information Files S2-S3 Files.
- The corresponding author provided re-analysis of the results using the revised code (S4 File).
- The following unsupported statements in the Abstract and Introduction were removed or revised, as indicated:
  - (Abstract) “*No algorithm has been developed that can detect SVs without control samples.*” This is inaccurate and has been removed.
  - (Introduction) “*HiC_breafinder [10] defined the SV breakpoints by searching the abnormal interaction block with higher interaction frequencies compared with the background model. More recently, the new method EagleC [11] combined deep-learning and ensemble-learning strategies to predict a full range of SVs. Both HiC_breakfinder and EagleC methods use several cell lines to construct a reference model that efficiently distinguishes intrachromosomal SV signals from other chromatin interactions.*” This statement has been revised in the final version.
  - (Introduction) “*These methods also cannot accurately predict the SVs of other species because of the variation in 3D genome organization features between species.*” This statement is inaccurate based on information in [3], and has been removed.

Two reviewers advised that the updated code functions as reported and that the code issues are resolved. Minor differences were identified for four SVs when comparing the results using the original and revised code which the corresponding author asserted do not affect the determination of true/false positive events or SV classifications of different lengths. The editors are satisfied that these differences do not impact the interpretation of the results.

In addition, concerns were raised that the evaluation of HiSV was not robust in [1] (uncorrected proof version), because when comparing the performance of HiSV against other tools, different binsize parameters were used for different tools. A member of the Editorial Board and a reviewer advised that HiSV is unable to predict SV below 50Kb resolution while HiC-breakfinder and Eagle C can predict SV at a higher resolution, and that HiC_breakfinder and EagleC were penalized more regarding window slack than HiSV. They requested additional analyses to improve the performance evaluation of HiSV in comparison with other tools.

The authors conducted additional analyses in efforts to address this issue, resulting in revisions to Figure 3, S1 File and the results for SV events by HiSV in each cancer cell line (provided in S4 File). In addition, the authors revised text in the Methods section titled, “Evaluation of SV callsets” and the Results sections titled, “Performance evaluating using simulation data” and “Performance evaluating using cancer cell lines.”

A member of the Editorial Board and a reviewer reviewed the updates and advised that concerns regarding the validity of the performance evaluation were not fully resolved. Specifically, they advised that the performance evaluation is still not sufficiently robust, and that the results reported in the updated version of the article do not support the claim that HiSV achieved a higher level of accuracy and sensitivity than existing methods. The corresponding author agreed that HiSV does not outperform HiC_breakfinder in some samples and revised the article to acknowledge this.

The *PLOS Computational Biology* Editors concluded that the method reported in [1] is a scientifically valid contribution to the literature, but that claims about its superiority to other methods are not adequately supported in light of the unresolved concerns about the performance evaluation. Therefore, we issue this Expression of Concern to inform readers of the unresolved issue, the concerns raised about the original publication, and updates made to the article [1] between the uncorrected proof version (published on January 6, 2023) and the final version of record (published on April 1, 2025).

The originally published, uncorrected proof and the final version of record are provided here for reference.

## Supporting information

S1 File Originally published, uncorrected proof version of article. Also available with the article [1] as Version 1 Uncorrected Proof.
(PDF)

S2 File Updated final version of record. Also available with the article [1] as Version 2.
(PDF)
