# Supplementary material for: HiSV: A control-free method for structural variation detection from Hi-C data
Source: PLoS Comput Biol. 2023 Jan 6;19(1):e1010760. doi: 10.1371/journal.pcbi.1010760 (PMC11960816; doi:10.1371/journal.pcbi.1010760)
Supplement: S1 File — Fig A. The precise and recall rates of SVs detected by HiSV and EagleC in MCF7 and HCC1954 cell line. Fig B. Size distribution of validated intra-chromosomal SVs detected by HiSV, HiC_breakfinder and EagleC. Fig C. Performance comparison of HiSV with existing methods in simulation samples. The F1-score is used to evaluate the sensitivity of HiSV and other methods to detect SVs in different tumor purity samples. Fig D. Performance comparison of HiSV with existing methods in cancer cell lines. The AUPR is used to evaluate the result of HiSV and other methods to detect SVs in different samples. Fig E. Performance comparison of HiSV and HiC_breakfinder in different SV types. Fig F. Comparison of SVs detected by HiSV and WGS methods. The detection results of WGS methods and HiSV for different types of SVs in K562 (a) and T47D (b). Fig G. The detection results of integrating WGS and Hi-C for different types of SVs. Fig H. Determination of SV breakpoints. The breakpoint was determined by searching the sign of the first eigenvector or principal component changes. Fig I. Classification of the different types of SVs. The cartoon in the box depicts the direction in which the interaction frequently decreases within the breakpoint region. Fig J. Suggested t of HiSV. We assessed the effect of bin size (a), sequencing depth (b) and assay (c) on the choice of parameter t. Fig K. The precise and recall rates of SVs detected at high resolution by HiSV, HiC_breakfinder and EagleC in MCF7 and HCC1954 cell line. (DOCX) [file pcbi.1010760.s001.docx]

Supplementary File

HiSV: a control-free method for structural variation detection from Hi-C data

Junping Li^1^, Lin Gao^1,*^ and Yusen Ye^1^

^1^ Department of Computer Science, School of Computer Science and Technology, Xidian University, Xi’an, Shaanxi 710071, China

* [lgao@mail.xidian.edu.cn](mailto:lgao@mail.xidian.edu.cn)

**Performance comparison of HiSV and EagleC**

We evaluated the performances of HiSV by comparing it with EagleC on MCF7 and HCC1954 cell lines. Different methods vary considerably in precision and recall rate. As shown in Fig A, we found that the F1-score of HiSV was significantly higher than EagleC for inter-chromosomal SVs. For example, the F1-scores of HiSV at MCF7 and HCC1954 were 31% and 20%, respectively, while the F1-scores of EagleC were 23% and 9%. However, EagleC had a higher F1-score than HiSV in detecting intra-chromosomal SVs.


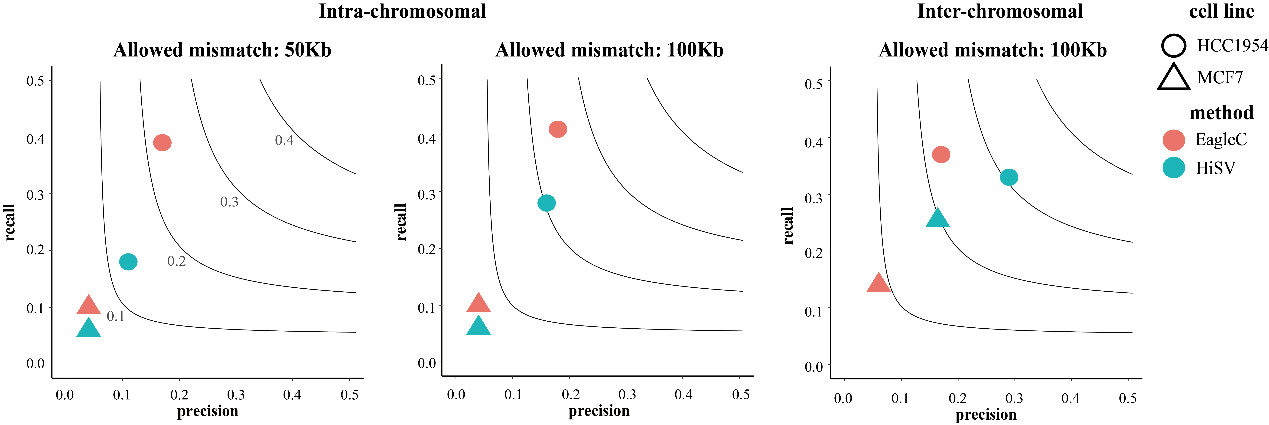


**Fig A.** The precise and recall rates of SVs detected by HiSV and EagleC in MCF7 and HCC1954 cell line.

**The range of SVs detection by HiSV, HiC_breakfinder and EagleC**

To determine the range of SVs detection by different methods, we counted the number of different sizes of validated intra-chromosomal SVs detected by HiSV, HiC_breakfinder and EagleC. It is worth mentioning that EagleC used K562 and T47D cell lines as training samples, so we excluded it when comparing the results of K562 and T47D cell lines. As shown in the Fig B, we found that EagleC can detect short-range SVs (<100Kb), while HiSV can detect a number of SVs smaller than 1Mb and HiC_breakfinder can only detect SVs greater than 1Mb. The lower bound of the range of SVs that HiSV, HiC_breakfinder and EagleC can identify in all three cell lines are 600Kb, 3.2Mb and 65Kb, respectively. Overall, these results confirmed that EagleC can distinguish real SVs from false-positive signals induced by normal 3D genome structures using a training model. And HiSV is a powerful tool that detects large-scale SVs from Hi-C data without control samples.

**Fig B.** Size distribution of validated intra-chromosomal SVs detected by HiSV, HiC_breakfinder and EagleC.

**
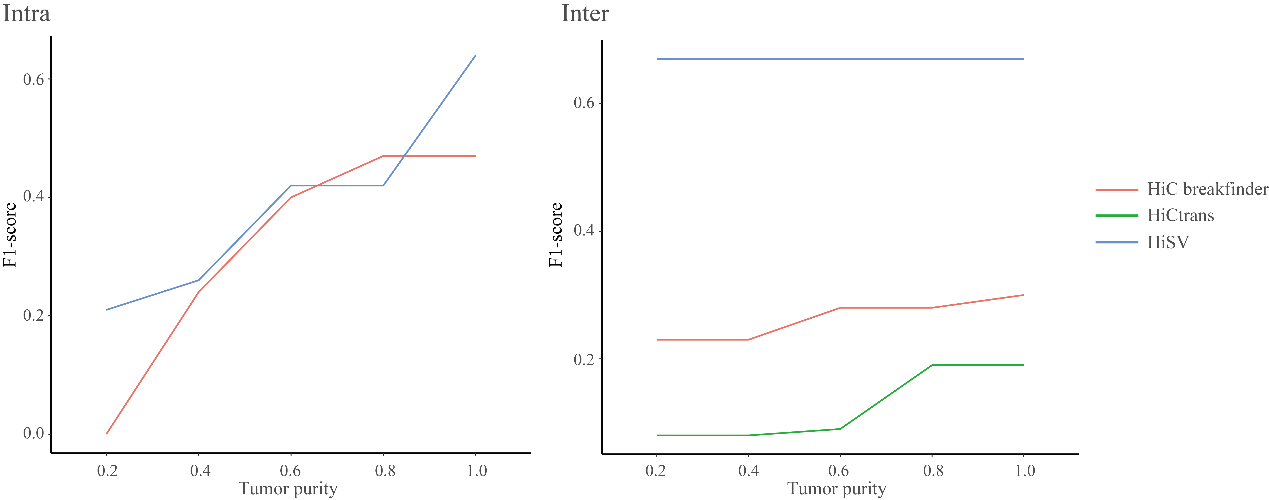
**

**Fig C. Performance comparison of HiSV with existing methods in simulation samples.** The F1-score is used to evaluate the sensitivity of HiSV and other methods to detect SVs in different tumor purity samples.

**
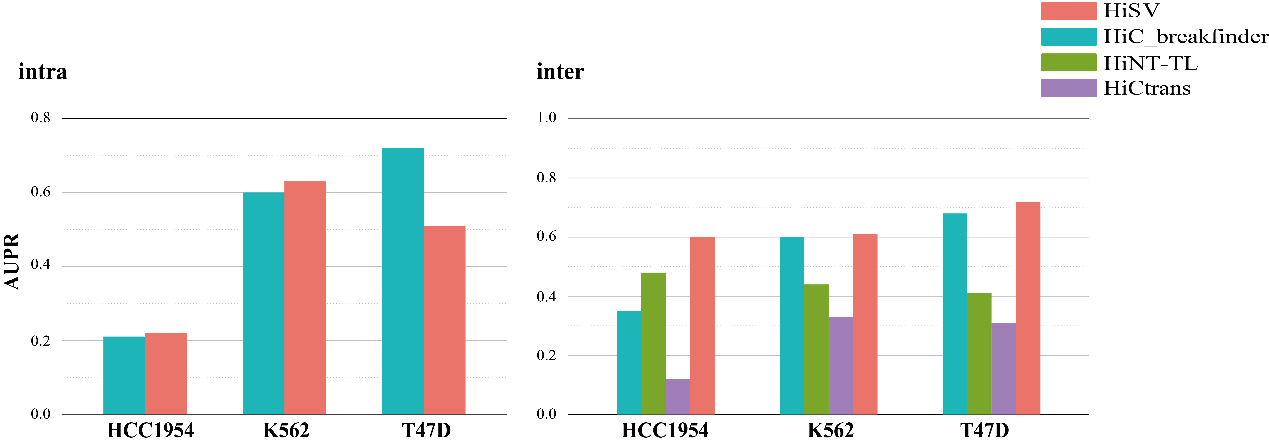
**

**Fig D.** **Performance comparison of HiSV with existing methods in cancer cell lines.** The AUPR is used to evaluate the result of HiSV and other methods to detect SVs in different samples.

**
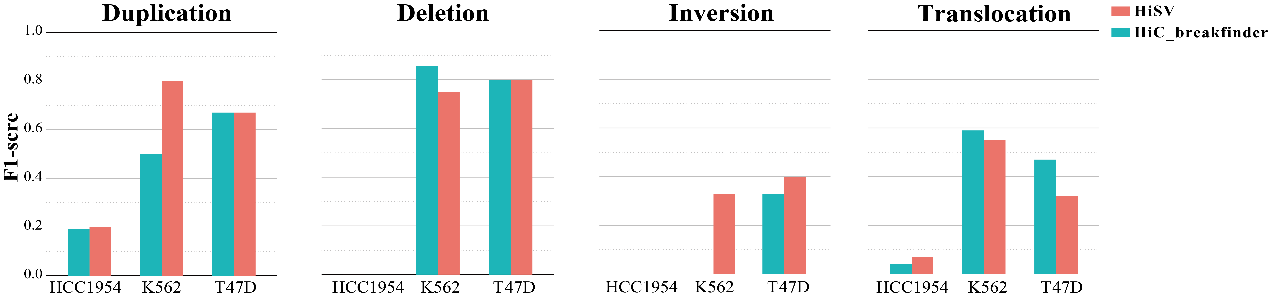
**

**Fig E.** Performance comparison of HiSV and HiC_breakfinder in different SV types.

**Fig F. Comparison of SVs detected by HiSV and WGS methods.** The detection results of WGS methods and HiSV for different types of SVs in K562 (a) and T47D (b).

**Fig G.** The detection results of integrating WGS and Hi-C for different types of SVs.

**Fig H.** **Determination of SV breakpoints.** The breakpoint was determined by searching the sign of the first eigenvector or principal component changes.

**Fig I.** **Classification of the different types of SVs.** The cartoon in the box depicts the direction in which the interaction frequently decreases within the breakpoint region.

**Suggested *t* of HiSV**

To assess the effect of bin size, sequencing depth and assay on the choice of parameter $t$, we constructed three sets of Hi-C data at different resolutions and sequencing depth: (a) We created Hi-C matrices at 25Kb, 50Kb and 100Kb resolutions, respectively; (b) We downsampled the original contact matrix (50Kb resolution) at different ratios (0.05, 0.1, 0.2, 0.4, 0.6, 0.8) to simulate Hi-C data at different sequencing depths; (c) We downsampled the original contact matrix (50Kb resolution) to simulate Hi-C data with the same sequencing depths as HiChIP data. We used the F1-score, the harmonic mean of the precision and recall rates, to evaluate the performance of HiSV at different parameter $t$. The results are shown in the figure below. We observed that HiSV has the highest F1-score when $t$ was set to 0.6 for different resolutions of Hi-C data. Furthermore, we found that HiSV achieved the best performance with $t$ set to 0.6 when sequencing depths approach or exceed 100 million reads ($ratio>0.1$). When the sequencing depth is less than 100 million reads ($ratio\leq0.1$), we achieve the best performance with $t$ set to 0.5. For different technologies, HiSV has the highest F1-score when was set to 0.6 for Hi-C data and set to 0.7 for HiChIP data. In conclusion, the parameter $t$ is not sensitive to the choice of bin size, but is sensitive to sequencing depth and sequencing technology. For Hi-C data with high sequencing depth (nearly 100 million contacts), we recommend setting $t$ to 0.6, and for low sequencing depth (less than 100 million contacts) we recommend setting $t$ to 0.5. For HiChIP data, we recommend setting $t$ to 0.7 to filter out more false-positive regions.

**Fig J.** **Suggested *t* of HiSV.** We assessed the effect of bin size (a), sequencing depth (b) and assay (c) on the choice of parameter *t*.

**The HiSV extension was applied to matched samples**

If matched tumor and normal pairs exist, firstly, we downsampled the high sequencing depth sample so that normal and tumor samples have the same sequencing depth. We then calculated the log2-ratio of interaction frequencies between the tumor and the normal samples for each bin pair. This ratio matrix is used as the input file for HiSV to detect SV events.

**Performance comparison of HiSV，HiC_breakfinder and EagleC at high resolution**

we polished the HiSV breakpoints to 10K and 5Kb resolution, and compared the results with HiC_breakfinder and EagleC respectively, which had the same resolution variable. As shown in Figure K, HiSV achieved a higher F1-score than HiC_breakfinder for all three cell lines. When increasing the resolution of HiSV from 50Kb to 5Kb, even though EagleC still outperformed HiSV in F1-score. the gap in F1-scores between EagleC and HiSV decreased from 9% to 6%.

**
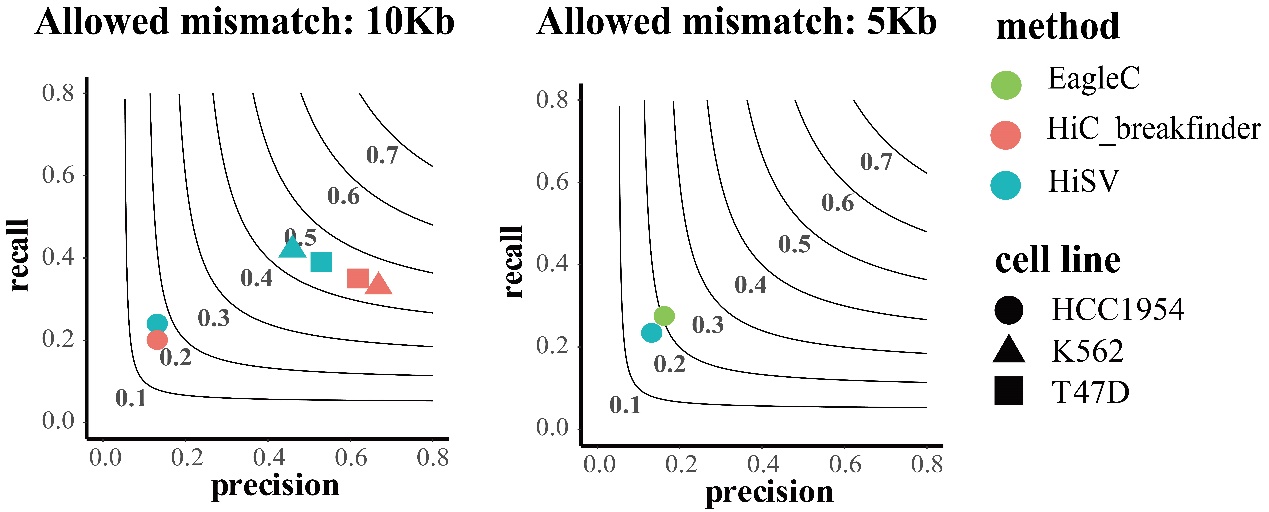
**

**Fig K.** The precise and recall rates of SVs detected at high resolution by HiSV, HiC_breakfinder and EagleC in MCF7 and HCC1954 cell line.
