# Supplementary material for: HiSV: A control-free method for structural variation detection from Hi-C data
Source: PLoS Comput Biol. 2023 Jan 6;19(1):e1010760. doi: 10.1371/journal.pcbi.1010760 (PMC11960816; doi:10.1371/journal.pcbi.1010760)
Supplement: Attachment — Submitted filename: Response to reviewers.docx [file pcbi.1010760.s006.docx]

**Dear editor and reviewers:**

Thanks so much for your time and efforts on our manuscript entitled “HiSV: a control-free method for structural variation detection from Hi-C data”. Also, we thank the reviewers for valuable comments and suggestions. These comments are valuable for revising and improving our manuscript. We have carefully revised our manuscript according to the reviewers’ suggestions and comments. Detailed responses to each reviewer are included on the accompanying page. The revised parts are highlighted in red in the new version of our manuscript. Also please let me know if there are remaining issues of concern. Thank you for your consideration.

**Reviewer #1:** The authors present HiSV (Hi-C for Structural Variation), an algorithm to find structural variants from Hi-C contact maps.

The advantages of HiSV include:
- it is control-free and works for more tissues or primary samples,
- it detects CNV, intra/inter-chrom translocations at the same time with a decision tree.

However, the evidence and experiments in this paper are not sufficient to prove HiSV is reliable. My detailed comments are as follows:

***1. There is not enough evidence to demonstrate that*** ***intra-chromosomal SVs can be separated from normal Hi-C contacts. Fig 1C shows that SV-related interactions are generally stronger than TADs. However, to make sure SVs can be separated from other structures:***

***1)*** ***SVs should be*** ***compared with the strongest TADs (Does “top 20 q_values” mean this in the figure caption? The authors should clarify.),***

***2) contact hot spots are not necessarily TADs, loops/stripes should also be considered.***

**Response:**

Thanks for your valuable comments. To demonstrate that large-scale intra-chromosomal SVs can be isolated from the Hi-C map, we first compared the significance of the interaction frequencies of stripes, TADs and SV boundaries on chromosome 4 in the K562 cell line. Furthermore, at over megabase distances, the expected interaction frequencies between two loci are close to zero, while interactions formed by large-scale SVs or TADs large than 1Mb are prominent in the surrounding region. Therefore, we then compared the significance of SV boundaries and strongest TADs large than 1Mb. The related results and discussion have been revised in the “Methods” section and Fig 1C. Details are given below.

We then compared the significance of the interaction frequencies of stripes, TADs and SV boundaries on chromosome 4 in the K562 cell line. The significances of interaction frequencies were calculated by FitHiC2 [16]. FitHiC2 corrects the resulting binomial p-values for the multiple testing using Benjamini-Hochberg to compute q-values, which represent the minimum false discovery rate (FDR). TAD boundaries were identified by Insulation Score [17] and stripes were identified by StripeCaller [18]. As shown in the Fig 1C, we observed that the interaction frequencies formed by SV events are more significant than most TADs and stripes, with a median q-value of 6.17e-165 for SV boundaries where 6.4e-5 for TADs and 3.8e-3 for stripes. We then counted the significance of TADs large than 1Mb. The minimum q-value of these TADs is 4.59e-8, which is greater than the median q-value of the SV boundary. Overall, we demonstrated that large-scale intra-chromosomal SVs can be isolated from the Hi-C map.

**Fig.1C** The significances of interaction frequencies of stripes, TADs and SV boundaries on chromosome 4 in K562 cell line.

**References in the revision:**

[16] Kaul A, Bhattacharyya S, Ay F. Identifying statistically significant chromatin contacts from Hi-C data with FitHiC2. Nat Protoc. 2020;15: 991–1012. doi:10.1038/s41596-019-0273-0

[17] Crane E, Bian Q, McCord RP, Lajoie BR, Wheeler BS, Ralston EJ, et al. Condensin-driven remodelling of X chromosome topology during dosage compensation. Nature. 2015;523: 240–244. doi:10.1038/nature14450

[18] Vian L, Pękowska A, Rao SSP, Kieffer-Kwon K-R, Jung S, Baranello L, et al. The Energetics and Physiological Impact of Cohesin Extrusion. Cell. 2018;173: 1165-1178.e20. doi:10.1016/j.cell.2018.03.072

***2. A predefined threshold is used to select SVs from other segments. How is the threshold chosen? Should it be dependent on map resolution, sequencing depth,*** ***or assay (Hi-C/capture Hi-C/Micro-C/…)? The authors should provide more information.***

**Response:**

Thanks for your valuable suggestions. We have added some experiments to explain how to choose the parameter $t$. Here, we used K562 cell line sequenced from different technologies, Hi-C (GSE63525) and HiChIP (GSM2705044), where the Hi-C data had high sequencing depth and contained nearly a billion contact counts. To assess the effect of bin size, sequencing depth and assay on the choice of parameter $t$, we constructed three sets data at different resolutions, sequencing depth and assays: (a) We created Hi-C matrices at 25Kb, 50Kb and 100Kb resolutions, respectively; (b) We downsampled the original contact matrix (50Kb resolution) at different ratios (0.05, 0.1, 0.2, 0.4, 0.6, 0.8) to simulate Hi-C data at different sequencing depths; (c) We downsampled the original contact matrix (50Kb resolution) to simulate Hi-C data with the same sequencing depths as HiChIP data. We used the F1-score, the harmonic mean of the precision and recall rates, to evaluate the performance of HiSV at different parameter $t$. The results are shown in the figure below. We observed that HiSV has the highest F1-score when $t$ was set to 0.6 for different resolutions of Hi-C data. Furthermore, we found that HiSV achieved the best performance with $t$ set to 0.6 when sequencing depths approach or exceed 100 million reads ($ratio>0.1$). When the sequencing depth is less than 100 million reads ($ratio\leq0.1$), we achieve the best performance with $t$ set to 0.5. For different technologies, HiSV has the highest F1-score when was set to 0.6 for Hi-C data and set to 0.7 for HiChIP data. In conclusion, the parameter $t$ is not sensitive to the choice of bin size, but is sensitive to sequencing depth and sequencing technology. For Hi-C data with high sequencing depth (nearly 100 million contacts), we recommend setting $t$ to 0.6, and for low sequencing depth (less than 100 million contacts) we recommend setting $t$ to 0.5. For HiChIP data, we recommend setting $t$ to 0.7 to filter out more false-positive regions. The related results and discussions have been included in Supplementary File 1.

***3. Another way to evaluate the performance of the “predefined threshold” is to change it and calculate the AUROC.*** ***If this is also possible for other baseline methods, AUROC provides a more reliable comparison.***

**Response:**

Thanks for your valuable suggestions. AUROC is a very robust evaluation metric but it is difficult to define negative events for cancer samples, so we cannot calculate the false positive rate of the detection result. Here, we used another reliable threshold-free metric, the area under the precision-recall curve (AUPR), to evaluate the performance of each method. We have evaluated the AUPR of each method in three cancer cell lines. As shown in the figure below, HISV performed very well across nearly all cell lines, with an average AUPR value of 0.62 for intra-chromosomal SVs and 0.63 for inter-chromosomal SVs across the three cell lines. The related results have been included in Supplementary Figure 4 and cited in the revised manuscript.

**S4 Fig.** **Performance comparison of HiSV with existing methods in cancer cell lines.** The AUPR is used to evaluate the result of HiSV and other methods to detect SVs in different samples.

***4. In Fig 3 (a and c), the precision and recall are calculated with all SVs. Is it possible to evaluate the performance in different SV types? If HiSV performs well in CNV, it also helps answer my questions in Comment 1.***

**Response:**

Thanks for your comments. We have evaluated the performance of HiSV and HiC_breakfinder in different SV types in the following figure. The related results have been included in the “Results” section and Supplementary Figure 5. Details are given below.

Then, we evaluated the performance of different SV types (S5 Fig). We found that HiSV has a consistently higher F1-score than HiC_breakfinder across nearly all SV types. For example, the F1-scores for HiSV to detect duplication in HCC1954 and K562 cell lines were 67% and 88% respectively, while the F1-scores for HiC_breakfinder were 55% and 46%.

**S5 Fig.** Performance comparison of HiSV and HiC_breakfinder in different SV types.

***5. From Fig 3c, I can see many false positive SVs called by HiSV, which might also be related to the limited separation I mentioned in Comment 1. What will we get if applying HiSV to a “normal cell”?***

**Response:**

Thanks for your comments. We have added some experiments to our work. First, we applied HiSV to GM12878 (lymphoblast) cell line and compared the result with the K562 (erythroleukemic) cell line. Here, we downsampled the GM12878 cell line to the same sequencing depth as the K562 cell line and retained the parameter settings of the K562 cell line ($binsize=50Kb,t=0.5$). As shown in Figure A, we observed that HiSV detected 1 SV and 47 SVs in GM12878 and K562 cell lines, respectively. There are no overlapping SVs in the two cell lines. These results indicated that these false-positive SVs are not associated with normal 3D genome structures.

But it is undeniable that we did detect many false positive SVs, especially in the HCC1954 cell line. To determine the causes of false positives in HCC1954, we counted the distribution of false-positive SVs by genomic position. We found that false positive SVs are concentrated on chromosomes 5, 9, 19 and 21 (Figure B). Therefore, we visualized the Hi-C maps of these chromosomes in Figure C. We noticed chromothripsis-like events in these chromosomes and the Hi-C maps showed considerable changes, these complex SV events have also been reported in [1]. HiSV detected abnormal interaction blocks in these Hi-C maps as an SV event, but these are not all reported in Groundtruth, so they are defined as false-positive SVs. Currently, HiSV cannot handle the chromothripsis event, but we hope that this issue can be addressed in our next study.

***6. The authors should proofread the paper to make sure concepts are well explained in the text and figure captions. For example,***

***1) the “top 20 q_values” in Comment 1,***

***2) “DEG” or “DGE”? The full name should be provided when the abbreviation is mentioned for the first time,***

***3) I think*** ***the “dist()” part in formula 3 is wrong.***

***4) the colors of the two types of translocations in Fig 6b are too similar to distinguish.***

**Response:**

Thanks for your suggestions. We respond to these four questions as follows.

1) We have added the definition of q_values in the manuscript as “FitHiC2 correct the resulting binomial p-values for the multiple testing using Benjamini-Hochberg to compute q-values, which represent the minimum false discovery rate (FDR).”.
2) We have added the full name of DEGs in the manuscript as “As shown in Fig 5A, a total of 2569 differentially expressed genes (DEGs) were detected between GM12878 and K562 cell lines.”. We also revised "DGE" into "DEG" in the full manuscript.
3) We have modified the manuscript as “”.
4) We have modified the colors of the two types of translocations in Fig 6b.

***7. Is it true that only “above threshold” pixels are chosen? Is it possible that some depleted contacts also correspond to SVs?***

**Response:**

Thanks for your comments. We did miss some true SV events when choosing the threshold. However, when we lowered the threshold, more false positives were detected despite obtaining high recalls. Here, we evaluate the performance of HiSV at different parameter $t$ in K562 cell line. As shown in the below figure, when we set $t$ to 0.2, although we detected almost all SV events, we reported a very higher number of false positive events with the precision of 0.1. Therefore, we set the best choice of $t$ that achieves the highest F1-score, which is defined as the harmonic mean of precision and recall.

***8. In Line 88, “the interaction frequencies between two loci decrease logarithmically with genome distance”. I think people usually use “power law” to describe the distance decay of contacts.***

**Response:**

Thanks for your suggestions. We have modified the sentence in the manuscript as “the interaction frequencies scales as a power law with genomic distance [15]”.

**References in the revision:**

15.	Wang S, Su J-H, Beliveau BJ, Bintu B, Moffitt JR, Wu C, et al. Spatial organization of chromatin domains and compartments in single chromosomes. Science. 2016;353: 598–602. doi:10.1126/science.aaf8084

***9. Can HiSV support other common Hi-C formats like .hic or .mcool?***

**Response:**

Thanks for your valuable suggestions, which have improved our work. We have added the Hi-C file in .hic, .cool and .mcool format as input. The code of this module can be accessed at: <https://github.com/GaoLabXDU/HiSV>. We also have revised this part of the description in the manuscript as “Our method supports raw fastq, bam file, .hic, .mcool and Hi-C Matrix files as its input.”

**Reviewer #2**: In this manuscript, Li et. al. present HiSV, a saliency-based method, which aims to identify structural variants from HiC data. Compared to other SV detection tools based on HiC, HiSV is control-free and achieved better performance on simulated data and cancer cell lines. In addition, HiSV can complement the SV identification from WGS methods. The manuscript is clearly written and well organized.

I have four major concerns.

***1. Calling the exact starting and ending position of structural variants can be challenging. It seems that HiSV c******ould only identify SV at bin pair level (for example 5kb, 10kb). What if one wants to find the exact location of a ~3kb SV?***

**Response:**

Thanks for your valuable suggestions, which have improved our work. We have added the module to re-determine the breakpoint. These descriptions have been supplemented as in the “Methods” section. Details are given below.

We first find SVs at a given bin size (eg.50Kb). After this initial phase, we further analyze the submatrix near the SV breakpoints with smaller binsize (eg.1Kb). As shown in S1 Fig, the breakpoints of SVs can be recognized by locating the change point in the submatrix. We performed PCA on the rows and columns of the matrix separately, and breakpoints were determined where the sign of the first eigenvector or principal component changes [21].

**S1 Fig.** Determination of SV breakpoints. The breakpoint was determined by searching the sign of the first eigenvector or principal component changes.

Furthermore, we compared the shifting distance between the detected breakpoint and ground truth in K562 and T47D cancer cell lines. As shown in Figure B, we observed that HiSV+PCA has the smallest shifting distance and achieved more accurate breakpoint detection than other methods. The related results have been included in Supplementary File 1.

**References in the revision:**

[21] Wang X, Xu J, Zhang B, Hou Y, Song F, Lyu H, et al. Genome-wide detection of enhancer-hijacking events from chromatin interaction data in rearranged genomes. Nat Methods. 2021;18: 661–668. doi:10.1038/s41592-021-01164-w

***2.*** ***HiC_breakfinder and EagleC use*** ***normal cell lines to construct background models. When normal cell lines are available, can*** ***HiSV*** ***incorporate this information to provide better identification of SV?***

**Response:**

Thanks for your valuable suggestion. When the control sample is available, HiSV can be easily extended to apply to matched samples and incorporate the information from the control sample to identify SVs. Here, we did not use normal cell lines to construct background models. A common obstacle in cancer genomes is the lack of an appropriate control sample for normal tissue [2]. Cell lines are a key tool in preclinical cancer research, but it is unclear to what extent they represent patient tumor samples [3], so the background model constructed with normal cell lines often does not work well with patient samples. Therefore, our goal is to develop a control-free method to detect SVs from Hi-C data.

If matched tumor and normal pairs exist, firstly, we downsampled the high sequencing depth sample so that normal and tumor samples have the same sequencing depth. We then calculated the log2-ratio of interaction frequencies between the tumor and the normal samples for each bin pair. This ratio matrix is used as the input file for HiSV to detect SV events. These descriptions have been included in Supplementary File 1.

***3. The authors exclude the comparison with EagleC since it is a supervised model. However, EagleC can be pre-trained and applied to 91 Hi-C datasets and 25 HiChIP/ChIA-PET datasets from 105 cancer cell lines or*** ***primary tumors. It should be a reasonable comparison to see how the pre-trained EagleC performs on the data used in this manuscript.***

**Response:**

Thanks for your suggestion. We have added the EagleC [4] method to our comparison. It is worth mentioning that EagleC used K562 and T47D cell lines as training samples, so we only compared the performance of HCC1954 cell line. First, we counted the number of different sizes of validated SVs detected by HiSV, HiC_breakfinder and EagleC. As shown in Figure A, we found that EagleC can detect smaller SVs (<100Kb), while HiSV can detect a small number of SVs smaller than 1Mb and HiC_breakfinder can only detect SVs greater than 1Mb. These results confirmed that EagleC can distinguish real SVs from false-positive signals induced by normal 3D genome structures using a training model. And HiSV can detect a more range of SVs than HiC_breakfinder without requiring control samples. Next, we evaluated the performance of EagleC in detecting large-scale SVs (>1Mb) and inter-chromosomal translocation. As shown in Figure B, we found that the F1-score of HiSV was significantly higher than EagleC for inter-chromosomal SVs but the F1-score of EagleC was one percentage point higher than that of HiSV in detecting intra-chromosomal SVs (0.47 VS 0.48). The related results have been included in Supplementary File 1.

***4. The ground truth is defined as SVs with lengths greater than 1MB. Can HiSV predict short-range (<1MB) SVs? How does it compare with other SV detection tools?***

**Response:**

Thanks for your suggestion. Hi-C maps contain features such as TAD and chromatin loops, typically less than 1Mb [5], and such patterns make the detection of short-range SVs challenging. Currently, HiSV cannot effectively predict short-range (<1Mb) SVs. However, our work does not ignore these short-range SVs. Previous studies have shown that whole-genome sequencing (WGS) can identify small SVs (>50 bp). The Hi-C technique provides higher chances than WGS to detect large-scale SVs in repetitive regions [6]. Therefore, we integrated the SV signals from HiSV and the WGS method to comprehensively identify SVs.

***5. Line 316 dist[(i,j),(p,q)] seems to be a typo. I think you are referring to dist(z_i,j - z_p,q).***

**Response:**

Thanks for your care and patience. This is our negligence. The “dist()” part in formula 3 is wrong. We have modified the manuscript as “$dist[(i,j),(p,q)]$”.

**Reviewer #3:** Li et al present an interesting computational method named HiSV to identify structure variations (SV) from the Hi-C contact map. HiSV out-perform existing unsupervised methods with the ability to detect both intra-chromosomal and inter-chromosomal SVs. Overall, I found the manuscript is reasonably clear and the use of the saliency map is the main novelty of this study. Despite the merit of this method, I have significant concerns about the robustness of the method towards known experimental biases and the choice of free parameters. My detailed comments are listed below.

Major comments:

***1.*** ***(a) In the introduction section, the authors claim that one advantage of HiSV over HiC_breakfinder and EagleC is that HiSV does not need a background model such that HiSV can be applied to tissue samples. First,*** ***this statement is not accurate.*** ***EagleC only requires a handful of negative samples from the normal cell lines for training. The authors of EagleC actually apply their method to over 100 Hi-C datasets from either cancer cell lines or primary tumors.*** ***HiNT-TL requires a background model, however, a generic background model averaged from multiple cell types also works well.***

***Response:***

Thanks for your comments and suggestions. We are very sorry that our statement in the manuscript is not accurate. Hi-C data contain features such as A/B compartments, TAD and chromatin loops, which make the detection of SVs challenging. Constructing a background model from the control sample or training a classifier from the matched sample (tumor and normal samples) are both effective ways to distinguish SV signals from other chromatin interactions. However, a common obstacle in cancer genomes is the lack of an appropriate control sample for normal tissue [2]. Cell lines are a key tool in preclinical cancer research, but it is unclear to what extent they represent patient tumor samples [3], so the reference model constructed with cell lines often do not work well with patient samples. Therefore, our goal is to develop a control-free method to detect SVs from Hi-C data. Based on the reviewer’s suggestion, we have modified the section in the manuscript as “HiC_breafinder [10] defined the SV breakpoints by searching the abnormal interaction block with higher interaction frequencies compared with the background model. More recently, the new method EagleC [11] combined deep-learning and ensemble-learning strategies to predict a full range of SVs. Both HiC_breakfinder and EagleC methods use several cell lines to construct a reference model that efficiently distinguishes intra-chromosomal SV signals from other chromatin interactions. However, a common obstacle in cancer genomes is the lack of an appropriate control sample for normal tissue [12]. Cell lines are a key tool in preclinical cancer research, but it is unclear to what extent they represent patient tumor samples [13], so the reference model constructed with cell lines often do not work well with patient samples. These methods also cannot accurately predict the SVs of other species because of the variation in 3D genome organization features between species [14].”

**References in the revision:**

10.	Dixon JR, Xu J, Dileep V, Zhan Y, Song F, Le VT, et al. Integrative detection and analysis of structural variation in cancer genomes. Nat Genet. 2018;50: 1388–1398. doi:10.1038/s41588-018-0195-8

11.	Wang X, Luan Y, Yue F. EagleC: A deep-learning framework for detecting a full range of structural variations from bulk and single-cell contact maps. Sci Adv. 2022;8: eabn9215. doi:10.1126/sciadv.abn9215

12.	Boeva V, Popova T, Bleakley K, Chiche P, Cappo J, Schleiermacher G, et al. Control-FREEC: a tool for assessing copy number and allelic content using next-generation sequencing data. Bioinformatics. 2012;28: 423–5. doi:10.1093/bioinformatics/btr670

13.	Mirabelli P, Coppola L, Salvatore M. Cancer Cell Lines Are Useful Model Systems for Medical Research. Cancers (Basel). 2019;11: E1098. doi:10.3390/cancers11081098

14.	Yang Y, Zhang Y, Ren B, Dixon JR, Ma J. Comparing 3D Genome Organization in Multiple Species Using Phylo-HMRF. Cell Syst. 2019;8: 494-505.e14. doi:10.1016/j.cels.2019.05.011

***(b) On the other hand,*** ***the usage of the background model is helpful to distinguish SVs from 3D genome features such as interactions between*** ***A/B compartments, especially SVs within a short distance (e.g., less than 1Mb).*** ***I wonder what is*** ***the lower bound of the range of SVs that HiSV can identify, because long-range intra-chromosomal SVs are similar to inter-chromosomal SVs while short-range intra-chromosomal SVs are much harder to identify.***

**Response:**

To determine the range of SVs detection by different methods, we counted the size distributions of validated intra-chromosomal SVs detected by HiSV, HiC_breakfinder and EagleC. It is worth mentioning that EagleC used K562 and T47D cell lines as training samples, so we excluded EagleC when comparing the results of K562 and T47D cell lines. As shown in the figure below, we found that EagleC can detect short-range SVs (<100Kb), while HiSV can detect a number of SVs smaller than 1Mb and HiC_breakfinder can only detect SVs greater than 1Mb. The lower bound of the range of SVs that HiSV, HiC_breakfinder and EagleC can identify in all three cell lines are 600Kb, 3.2Mb and 65Kb, respectively. Overall, these results confirmed that EagleC can distinguish real SVs from false-positive signals induced by normal 3D genome structures using a training model. And HiSV is a powerful tool that detects large-scale SVs (>1Mb) from Hi-C data without control samples. The related results have been included in Supplementary File 1.

***(C)*** ***The authors should provide a comprehensive assessment of the capability of HiSV in detecting various size ranges of intra-chromosomal SVs.*** ***For example, the authors should add more groups (e.g., < 1Mb) in Figure 3D and*** ***add a category of SVs that were missed by HiSV but appeared in the ground truth.*** ***Simulation results would also be quite helpful to demonstrate the capability of HiSV.***

**Response:**

We then compared the number of missed intra-chromosomal SVs by HiSV and HiC_breakfinder. Here, we classified the ground truth into four categories according to their length, namely ≤1Mb, ≤2Mb, ≤5M and > 5M SVs. The simulation data contained 24 SVs of length 500Kb-2Mb and the lower bound of ground truth for cancer cell lines is 500Kb. As shown in the figure below, we observed that HiSV detected more ≤ 5Mb SVs than HiC_breakfinder. But both methods missed most short-range SVs (≤1Mb) in simulation data and cancer cell lines.

However, our work does not ignore these short-range SVs. Previous studies have shown that whole-genome sequencing (WGS) can identify small SVs (>50 bp). Hi-C technique provides higher chances than WGS to detect large-scale SVs in repetitive regions [6]. Therefore, we integrated the SV signals from HiSV and the WGS method to comprehensively identify SVs.

***2. The authors should provide more explanation on how to choose parameter t.*** ***For example, given what kind of sequence depth, should we choose 0.5 rather than 0.6? The authors may consider down-sampling a Hi-C dataset and run HiSV under different values of t and*** ***assess what would be the best choice of t given different sequencing depths. In addition, it is unclear if parameter t is sensitive to the choice of bin size.***

**Response:**

Thanks for your valuable suggestions. We have added some experiments to explain how to choose the parameter $t$. Here, we used K562 cell line with high sequencing depth, which contains nearly a billion contact counts (GSE63525). To assess the effect of bin size and sequencing depth on the choice of parameter $t$, we constructed two sets of Hi-C data at different resolutions and sequencing depth: (a) We created Hi-C matrices at 25Kb, 50Kb and 100Kb resolutions, respectively. (b) We downsampled the original contact matrix (50Kb resolution) at different ratios (0.05, 0.1, 0.2, 0.4, 0.6, 0.8) to simulate Hi-C data at different sequencing depths. We used the F1-score, the harmonic mean of the precision and recall rates, to evaluate the performance of HiSV at different parameter $t$. The results are shown in the figure below. We observed that HiSV has the highest F1-score when $t$ was set to 0.6 for different resolutions of Hi-C data. Furthermore, we found that HiSV achieved the best performance with $t$ set to 0.6 when sequencing depths approach or exceed 100 million reads ($ratio>0.1$). When the sequencing depth is less than 100 million reads ($ratio\leq0.1$), we achieve the best performance with $t$ set to 0.5. In conclusion, the parameter $t$ is not sensitive to the choice of bin size, but is sensitive to sequencing depth. For Hi-C data with high sequencing depth (nearly 100 million contacts), we recommend setting $t$ to 0.6, and for low sequencing depth (less than 100 million contacts) we recommend setting $t$ to 0.5. The related results and discussions have been included in Supplementary File 1.

***3. The authors should take Hi-C biases such as GC content, the number of restriction sites, and mappability into account when calculate the average profiles, which is used to annotate SVs into different types.***

**Response:**

Thanks for your valuable suggestions, which have improved our work. We did not consider the effect of these biases on the average profiles. So, we have added a generalized additive model proposed by HiNT [7] to correct the bias in GC content, mappability, and the number of restriction sites. We also have revised this part of the description in the manuscript as “First, we computed a one-dimensional coverage profile at each bin across the genome. We then employed a generalized additive model [9] on the coverage profile to correct the bias in GC content, mappability, and the number of restriction sites”.

**References in the revision:**

9.	Wang S, Lee S, Chu C, Jain D, Kerpedjiev P, Nelson GM, et al. HiNT: a computational method for detecting copy number variations and translocations from Hi-C data. Genome Biol. 2020;21: 73. doi:10.1186/s13059-020-01986-5

***4. The authors should add a description of the output file in the GitHub repo.***

**Response:**

Thanks for your suggestion. We have added the detailed description for the output file in GitHub (<https://github.com/GaoLabXDU/HiSV>.).

***5. The authors should explain the definition of q_values in the caption of Figure 1C***

**Response:**

Thanks for your suggestion. We have added the definition of q_values in the manuscript as “FitHiC2 correct the resulting binomial p-values for the multiple testing using Benjamini-Hochberg to compute q-values, which represent the minimum false discovery rate (FDR).”.

***6. Line 73, typo "******specie samples”***

**Response:**

Thanks for your care and patience. This is our negligence. We have revised “specie samples” in the manuscript as “species”.

**Reference**

1.	Akdemir KC, Le VT, Chandran S, Li Y, Verhaak RG, Beroukhim R, et al. Disruption of chromatin folding domains by somatic genomic rearrangements in human cancer. Nat Genet. 2020;52: 294–305. doi:10.1038/s41588-019-0564-y

2.	Boeva V, Popova T, Bleakley K, Chiche P, Cappo J, Schleiermacher G, et al. Control-FREEC: a tool for assessing copy number and allelic content using next-generation sequencing data. Bioinformatics. 2012;28: 423–5. doi:10.1093/bioinformatics/btr670

3.	Mirabelli P, Coppola L, Salvatore M. Cancer Cell Lines Are Useful Model Systems for Medical Research. Cancers (Basel). 2019;11: E1098. doi:10.3390/cancers11081098

4.	Wang X, Luan Y, Yue F. EagleC: A deep-learning framework for detecting a full range of structural variations from bulk and single-cell contact maps. Sci Adv. 2022;8: eabn9215. doi:10.1126/sciadv.abn9215

5.	Gong Y, Lazaris C, Sakellaropoulos T, Lozano A, Kambadur P, Ntziachristos P, et al. Stratification of TAD boundaries reveals preferential insulation of super-enhancers by strong boundaries. Nat Commun. 2018;9: 542. doi:10.1038/s41467-018-03017-1

6.	Wu P, Li T, Li R, Jia L, Zhu P, Liu Y, et al. 3D genome of multiple myeloma reveals spatial genome disorganization associated with copy number variations. Nat Commun. 2017;8: 1937. doi:10.1038/s41467-017-01793-w

7.	Wang S, Lee S, Chu C, Jain D, Kerpedjiev P, Nelson GM, et al. HiNT: a computational method for detecting copy number variations and translocations from Hi-C data. Genome Biol. 2020;21: 73. doi:10.1186/s13059-020-01986-5
